# American Indian and Hispanic Populations Have Cultural Values and Issues Similar to Those of Appalachian Populations

**Published:** 2007-06-15

**Authors:** Catherine A Marshall

**Affiliations:** Research Professor, Department of Educational Psychology, Northern Arizona University, Flagstaff, Ariz

## To the Editor:

I want to thank you and the Centers for Disease Control and Prevention for disseminating such important work regarding my home region of Appalachia (October 2006 issue of *Preventing Chronic Disease* ). It is based on the strength and support of my own southern Appalachian culture that I have been able to appreciate and support culturally focused interventions among the American Indian and Hispanic populations with whom I have worked during the past 25 years.

It is important to think of making linkages among peoples and regions to improve both science and interventions for low-income and underserved people facing cancer. For instance, between such seemingly different areas as southern Arizona and southern Appalachia there are similar values in terms of a focus on family, religion, and home, and there are similar problems among people with low socioeconomic status (SES) (e.g., lack of access to health care, low educational attainment). All of these aspects influence cancer intervention. Focusing on cancer in the Appalachian region, Behringer and Friedell (1) advised:

Focus group and survey participants reported that they gain most of their information about cancer from family, neighbors, and friends rather than from health professionals. Unfortunately, the information they receive often includes misperceptions of and dated knowledge about cancer treatments. The goal in Appalachia is to improve public cancer education while acknowledging and effectively using prevailing patterns of communication.

These same findings would not be surprising in research conducted with American Indian or Hispanic populations.

The Appalachian culture is, by definition it seems, linked to low SES and to poverty (2,3). In their article "Rehabilitation Counseling in Appalachian America," Bauer and Growick (4) noted, "In Appalachian America, income rates are lower, the unemployment is higher, poverty is rampant. ... [and] social and health services are scarce and scattered." These authors also suggested that a counselor should expect that a family member might attend a counseling session along with the individual who has a chronic illness or disability, commenting, "It is very important to Appalachian Americans to involve the entire family in the decision-making process; intergenerational support is an integral part of the fabric of life in Appalachia."

Low SES, too often synonymous with life in Appalachia, figures largely in cancer, in poor health in general (3,5,6), and in overall life expectancy (7). "Poor Americans, irrespective of race, have a 10% to 15% decreased rate of survival from cancer compared to the general population" (8). A study by Leybas-Amedia et al (9) noted that access to health care services and, in particular, cancer screening is more likely an issue of SES rather than culture.

Cancer, for individuals and families who are both poor and from underserved populations, can be an intimidating illness in terms of screening and a cost-prohibitive illness in terms of treatment. What better way to address cancer-related health disparities, and health disparities in general, than documenting and disseminating information and acting upon our common concerns and shared values.
